# Supplementary material for: Heat adaptation of phage T7 under an extended genetic code
Source: Virus Evol. 2021 Dec 1;7(2):veab100. doi: 10.1093/ve/veab100 (PMC8923235; doi:10.1093/ve/veab100)
Supplement: veab100_Supp [file veab100_supp.zip › Sequencing_Analysis.zip!/Sequencing_Analysis/S1/evidence/SNP_5.html]

BRESEQ :: Evidence


| Predicted mutation | | | | | | |
| --- | --- | --- | --- | --- | --- | --- |
| evidence | seq id | position | mutation | annotation | gene | description |
| RA | NC\_001604.1 | 21,265 | A→C | intergenic (–/–) | – / – | –/– |

| Read alignment evidence... | | | | | | | | | | | |
| --- | --- | --- | --- | --- | --- | --- | --- | --- | --- | --- | --- |
|  | seq id | position | | ref | new | freq | score (cons/poly) | reads | annotation | genes | product |
| \* | NC\_001604.1 | 21,265 | 0 | A | C | 91.4% | 12404.9 / inf | 4499 | intergenic (–/–) | *–/–* | –/– |
| Reads supporting (aligned to +/- strand):  **ref** base A (193/192);  **new** base C (2070/2027);  **total** (2272/2227) | | | | | | | | | | | |
| Fisher's exact test for biased strand distribution *p*-value = 9.15e-01 | | | | | | | | | | | |
| Kolmogorov-Smirnov test that lower quality scores support variant *p*-value = 9.91e-01 | | | | | | | | | | | |

Only 100 of 4508 total aligned reads displayed.  

`GACTTATCCTAAAGAGGCTTGCCCATACATCCCGATTCGGATGGTCAGACTAGATGGTGAATCCTACGGTCGTTCGTACATTGAGGAATACTTAGGTGACTTACGGTCCCTTGAAAATCTCCAAGAGGCTATCGTCAAGATGTCCATGATTAGCTCTAAGGTTATCGGCTTAGTGAATCCTGCTGGTATCACCCAGCCACGCCGACTGACCAAAGCTCAGACTGGTGACTTCGTTACTGGTCGTCCAGAAGACATCTCGTTCCTCCAACTGGAGAAGCAAGCAGACTTTACTGTAGCTAAAGCCGTAAGTGACGCTATCGAGGCTCGCCTTTCGTTTGCCTTTATGTTGAACTCTGCGGTTCAGCGTACAGGTGAACGTGTGACCGCCGAAGAGATTCGGTATGTAGCTTCTGAACTTGAAGATACTTTAGGTGG‑TGTCTACTCTATCCTTTCTCAAGAATTACAATTGCCTCTGGTACGAGTG‑CTCTTGAAGCAACTACAAGCCACGCAACAGATTCCTGAGTTACCTAAGGAAGCCGTAGAGCCAACCATTAGTACAGGTCTGGAAGCAATTGGTCGAGGACAAGACCTTGATAAG  >  NC_001604.1/20965‑21562`  

                                                                                                                                                                                                                                                                                                            `|`                                                                                                                                                                                                                                                                                                             
     `aTCCTAAAGAGGCTTGCCCATACATCCCGATTCGGATGGTCAGACTAGATGGTGAATCCTACGGTCGTTCGTACATTGAGGAATACTTAGGTGACTTACGGTCCCTTGAAAATCTCCAAGAGGCTATCGTCAAGATGTCCATGATTAGCTCTAAGGTTATCGGCTTAGTGAATCCTGCTGGTATCACCCAGCCACGCCGACTGACCAAAGCTCAGACTGGTGACTTCGTTACTGGTCGTCCAGAAGACATCTCGTTCCTCCAACTGGAGAAGCAAGCAGACTTTACTGTAGCTAACGCCGt                                                                                                                                                                                                                                                                                                        >  1:9115/1‑301 (MQ=255)`  
     `aTCCTAAAGAGGCTTGCCCATACATCCCGATTCGGATGGTCAGACTAGATGGTGAATCCTACGGTCGTTCGTACATTGAGGAATACTTAGGTGACTTACGGTCACTTGAAAATCTCAAAGAGGCTATCGTCAAGATGTCCATGATTAGCTCTAAGGTTATCGGCTTAGTGAATCCTGCTGGTATCACCCAGCCACGCCGACTGACCAAAGCTCAGACTGGTGACTTCGTTACTGGTAGTCCAGAAGCCATATCGTTCCTCCAACTGGAGAAGAAAGCAGACTTTACTGTAGCTCACGCCGt                                                                                                                                                                                                                                                                                                        >  1:305391/1‑301 (MQ=255)`  
        `cTAAAGAGGCTTGCCCATACATCCCGATTCGGATGGTCAGACTAGATGGTGAATCCTACGGTCGTTCGTACATTGAGGAATACTTAGGTGACTTACGGTCCCTTGAAAATCTCCAAGAGGCTATCGTCAAGATGTCCATGATTAGCTCTAAGGTTATCGGCTTAGTGAATCCTGCTGGTATCACCCAGCCACGCCGACTGACCAAAGCTCAGACTGGTGACTTCGTTACTGGTCGTCCAGACGACATCTCGTTCCTCCAACTGGAGAAGCAAGCAGACTTTACTGTAGCTAACGCCGTAAg                                                                                                                                                                                                                                                                                                     >  1:378961/1‑301 (MQ=255)`  
        `cTAAAGAGGCTTGCCCATACATCCCGATTCGGATGGTCAGACTAGAGGGTGAATCCTACGGTCGTTCGTACATTGAGGAATCCTTAGGTGACTTACGGTCCCTTGAAAATCTCCAAGAGGCTATCGTCAAGATGTCCATGATTAGCTCTAAGGTTATCGGCTTAGTGAATCCTGCTGGTATCACCCAGCCACGCCGACTGACCAAAGCTCAGACTGGTGACTTCGTTACTGGTCGTCCAGAAGACATCTCGTTCCTCCAACTGGAGAAGCAAGCAGACTTTACTGTAGCTAACGCCGTAAg                                                                                                                                                                                                                                                                                                     <  1:260707/301‑1 (MQ=255)`  
                       `cATACATCCCGATTCGGATGGTCAGACTAGATGGTGAATCCTACGGTCGTTCGTACATTGAGGAATACTTAGGTGACTTACGGTCCCTTGAAAATCTCCAAGAGGCTATCGTCAAGATGTCCATGATTAGCTCTAAGGTTATCGGCTTAGTGAATCCTGCTGGTATCACACAGACAAGCCGACTGACAAAAGCTCAGACTGGTGACTTCGTTAATGGTAGTCCAGAAGACATCTCGTTCCTCCAAATGTAGAAGCAAGCAGACTTTACTGTAGCTAAAGCAGTAAGTGACGCTATCGCGGc                                                                                                                                                                                                                                                                                      >  1:187326/1‑301 (MQ=255)`  
                        `aTACATCCCGATTCGGATGGTCAGACTAGATGGTGAATCCTACGGTCGTTCGTACATTGAGGAATACTTAGGTGACTTACGGTCCCTTGAAAATCTCCAAGAGGCTATCGTCAAGATGTCCATGATTAGCTCTAAGGTTATCGGCTTAGTGAATCCTGCTGGTATCACCCAGCCACGCCGACTGACCAAAGCTCAGACTGGTGACTTCGTTACTGGTCGTCCAGAAGACATCTCGTTCCTCCAACTGGAGAAGCAAGCAGACTTTACTGTAGCTAACGCCGTAAGTGACGCTATCGAGGCt                                                                                                                                                                                                                                                                                     <  1:174528/301‑1 (MQ=255)`  
                                   `ttCGGATGGTTAGACTAGATGGTTAATCCTACGTGCGGTCGTACATTGAGGAATACTTAGGTGACTTACGGTCCCTTGAAAATCTCCAAGAGGCTATCGTCAAGATGTCCATGATTAGCTCTAAGGTTATCGGATTAGTGAATCCTGCTGGTATCACCCAGCCACGCCGACTGACCAAAGCTCAGACTGGTGACTTCGTTACTGGTCGTCCAGAAGACATCTCGTTCCTCCAACTGGAGAAGCAAGCAGACTTTACTGTAGCTAACGCCGTAAGTGACGCTATCGAGGCTCGCCTTTCGtt                                                                                                                                                                                                                                                                          <  1:62355/301‑1 (MQ=255)`  
                                          `ggTCAGACTAGATGGTGAATCCTACGGTCGTTCGTACATTGAGGAATACTTAGGTGACTTACGGTCCCTTGAAAATCTCCAAGAGGCTATCGTCAAGATGTCCATGATTAGCTCTAAGGTTATCGGCTTAGTGAATCCTGCTGGTATCACCCAGCCAAGCCGACTGACCAAAGCTCAGACTGGTGACTTCGTTACTGGTCGTCCAGAAGACATCTCGTTCCTCCAACTGGAGAAGCAAGCAGACTTTACTGTAGCTAACGCCGTAAGGAACGCTATCGAGGCTCGCCTTTCGTTTGCCtct                                                                                                                                                                                                                                                                   >  1:550750/1‑299 (MQ=255)`  
                                               `gtcTAGATGGTGAATCCTACGGTCGTTCGTACATTGAGGAATACTTAGGTGACTTACGGTCCCTTGAAAATCGCCAAGAGGCTATCGTCAAGATGTCCATGATTAGATCTAAGGTTATCGGCTTAGTGAATCCTGCTGGTATCAACCAGCCACGCCGACTGACCAAAGCTCAGACTGGTGACTTCGTTACTGGTCGTCCAGAAGAAATCTCGTTCCTCCAACTGGAGAAGCAAGCAGACTTTACTGTAGCTAACGCCGTAAGTGACGCTATCGCGGCTCGCCTTTCGTTTGCCTTTATGtt                                                                                                                                                                                                                                                              >  1:234767/3‑301 (MQ=255)`  
                                                   `aGATGGTGAATCCTACGGTCGTTCGTACATTGAGGAATACTTAGGTGACTTACGGTCCCTTGAAAATCTCCAAGAGGCTATCGTCAAGATGTCCATGATTAGCTCTAAGGTTATCGGCTTAGTGAATCCTGCTGGTATCACCCAGCCACGCCGACTGACCAAAGCTCAGACTGGTGACTTCGTTACTGGTCGTCCAGAAGACATCTCGTTCCTCCAACTGGAGAAGCAAGCAGACTTTACTGTAGCTAACGCCGTAAGTGACGCTATCGCGGCTCGCCTTTCGTTTGCCTTTATGTTGAAc                                                                                                                                                                                                                                                          <  1:570992/301‑1 (MQ=255)`  
                                                   `aGATGGTGAATCCTACGGTCGTTCGTACATTGAGGAATACTTAGGTAACTTACGGTCCCTTGAAAATCTCCAAGAGGCTATCGTCAAGATGTCCATGATTAGCTCTAAGGTTATCGGCTTAGTGAATCCTGCTGGTATCACCCAGCCACGCCGACTGACCAAAGCTCAGACTGGTGACTTCGTTACTGGTCGTCCAGAAGACATCTCGTTCCTCCAACTTGAGAAGCAAGCAGACTTTACTGTAGCTAACGCCGTAAGTGACGCTATCGAGGCTCGCCTTTCGTTTGCCTTTATGTTGAAc                                                                                                                                                                                                                                                          <  1:473080/301‑1 (MQ=255)`  
                                                        `gTGAATCCTACGGTCGTTCGTACATTGAGGAATACTTAGGTGACTTACGGTCCCTTGAAAATCTCCAAGAGGCTATCGTCAAGATGTCCATGATTAGCTCTAAGGTTATCGGCTTAGTGAATCCTGCTGGTATCACCCAGCCACGCCGACTGACCAAAGCTCAGACTGGTGACTTCGTTACTGGTCGTCCAGAAGACATCTCGTTCCTCCAACTGGATAAGCAAGCAGACTTTACTGTAGCTAACCCCGTACGTGACGCTATCGCGGCTCGCCTTTCGTTTGCCTTTATGTTGTACTCTGc                                                                                                                                                                                                                                                     >  1:517405/1‑301 (MQ=255)`  
                                                          `gAATCCTACGGTCGTTCGTACATTGAGGAATACTTAGGTGACTTACGGTCCCTTGAAAATCTCCAAGAGGCTATCGTCAAGATGTCCATGATTAGCTCTAAGGTTATCGGCTTAGTGAATCCTGCTGGTATCACCCAACCACGCCGACTGACCAAAGCTCAGACTGGTGACTTCGTTACTGGTCGTCCAGAAGACATCTCGTTCCTCCAACTGGAGAAGCAAGCAGACTTTACTGTAGCTAACGCCGTAAGTGACGCTATCGAGGCTCGCCTTTCGTTTGCCTTTATGTTGAACTCTGCgg                                                                                                                                                                                                                                                   >  1:292421/1‑301 (MQ=255)`  
                                                                  `cGGTCGTTCGTACATTGAGGAATACTTAGGTGACTTACGGTCCCTTGAAAATCTCCAAGAGGCTATCGTCAAGATGTCCATGATTAGCTCTAAGGTTATCGGCTTAGTGAATCCTGCTGGTATCACCCAGCCACGCCGACTGACCAAAGCTCAGACTGGTGACTTCGTTACTGGTCGTCCAGAAGACATCTCGTTCCTCCAACTGGAGAAGCAAGCAGACTTTACTGTAGCTAACGCCGTAAGTGACGCTATCGCGGCTCGCCTTTCGTTTGCCTTTATGTTGAACTCTGCGGTTCAGCGt                                                                                                                                                                                                                                           <  1:546067/301‑1 (MQ=255)`  
                                                                    `ttcgttcgtAGATGGTGGAATACTTAGGTGACGTACGGTACCTTTGAATGCTCCAAGAGGCTATCGTCAATTTGTCCATGATTAGCTCTAAGGTTATCGGCTTAGTGAATCCTGCTGGTATCACCCAGCCACGCCGACTGGCCAAAGCTCAGACTGGTGACTTCGTTACTGGACGTCCAGAAGACATCTCGTTCCTCCAACTGGAGAAGCAAGCAGACTTTACTTTAGCTAACGCCGTAAGTGACGCTATCGAGGCTCGCCTTTCGTTTGCCTTTATGTTGAACTCTGCGGTTCAGAGTAc                                                                                                                                                                                                                                         <  1:145270/300‑1 (MQ=255)`  
                                                                      `cgttcgtACATTGAGGAATACTTAGGTGACTTACGGTCCCTTGAAAATCTCCAAGAGGCTATCGTCAAGATGTCCATGATTAGCTCTAAGGTTATCGGCTTAGTGAATCCTGCTGGTATCACCCAGCCACGCCGACTGACCAAAGCTCAGACTGGTGACTTCGTTACTGGTCGTCCAGAAGACATCTCGTTCCTCCAACTGGAGAAGCAAGCAGACTTTACTGTAGCTAACGCCGTAAGTGACGCTATCGCGGCTCGCCTTTCGTTTGCCTTTATGTTGAACTCTGCGGTTCAGCGTACAg                                                                                                                                                                                                                                       >  1:577743/1‑301 (MQ=255)`  
                                                                       `ggagatgtgtataagagacagtAGGTGACTTACGGTCCCTTGAAAATCTCCAAGAGGCTATCGTCAAGATGTCCATGATTAGCTCTAAGGTTATCGGCTTAGTGAATCCTGCTGGTATCACCCAGCCACGCCGACTGACCAAAGCTCAGACTGGTGACTTCGTTACTGGTCGTCCAGAAGACATCTCGTTCCTCCAACTGGAGAAGCAAGCAGACTTTACTGTAGCTAACGCCGTAAGTGACGCTATCGCGGCTCGCCTTTCGTTTGCCTTTATGTTGAACTCTGCGGTTCAGCGTACAgg                                                                                                                                                                                                                                      <  1:420133/280‑1 (MQ=255)`  
                                                                        `ttcgtACATTGAGGAATACTTAGTTGACTTACGGTCCCTTGAAAATCTCCAAGAGGCTATCGTCAATATGTCCATGATTAGCTCTAAGGTTATCGGCTTATTGAATCCTGCTGTTATCACCCAGCCACGCCGACTGACCAAAGCTCAGACTGGTGACTTCGTTACTGGTCGTCCAGAAGACATCTCGTTCCTCCAACTGGATAAGCAAGCAGACTTTACTGTAGCTAACGCCGTACTTGACGCTATCGCGGCTCCCCTTTCGTTTGCCTTTATGTTGAACTCTGCGGTTCAGCGTACAGGt                                                                                                                                                                                                                                     >  1:318045/1‑301 (MQ=255)`  
                                                                        `ttcgtACATTGAGGAATACTTAGGTGACTTACGGTCCCTTGAAAATCTCCAAGAGGCTATCGTCAAGATGTCCATGATTAGCTCTAAGGTTATCGGCTTAGTGAATCCTGCTGGTATCACCCAGCCACGCCGACTGACCAAAGCTCAGACTGGTGACTTCGTTACTGGTCTTCCAGAAGACATCTCGTTCCTCCAACTGGAGAAGCAAGCAGACTTTACTGTAGCTAACGCCGTAATTGACGCTATCGAGGCTCGCCTTTCGTTTGCCTTTATGTTGAACTCTGCGGTTCAGCGtctgtct                                                                                                                                                                                                                                     >  1:396831/1‑295 (MQ=255)`  
                                                                        `ttcgtACATTGAGGAATACTTAGGTGACTTACGGTCCCTTGAAAATCTCCAAGAGGCTATCGTCAAGATGTCCATGATTAGCTCTAAGGTTATCGGCTTAGTGAATCCTGCTGGTATCACCCAGCCACGCCGACTGACCAAAGCTCAGACTGGTGACTTCGTTACTGGTCGTCCAGAAGACATCTCGTTCCTCCAACTGGAGAAGCAAGCAGACTTTACTGTATCTAACGCCGTAAGTGACGCTATCGCGGCTCGCCTTTCGTTTGCCTTTATGTTGTACTCTTCGGTTcctgtctcttat                                                                                                                                                                                                                                     >  1:273202/1‑290 (MQ=255)`  
                                                                        `ttcgtACATTGAGGAATACTTAGGTGACTTACGGTCCCTTGAAAATCTCCAAGAGGCTATCGTCAAGATGTCCATGATTAGCTCTAAGGTTATCGGCTTAGTGAATCCTGCTGGTATCACCAAGCCACGCCGACTGACCAAAGCTCAGACTGGTGACTTCGTTACTGGTCGTCCAGAAGACATCTCGTTCCTCCAACTGGAGAAGCAAGCAGACTTTACTGTAGCTAACGCCGTAAGTGACGCTATCGCGGCTCGCCTTTCGTTTGCCTTTATGTTTAACTCTGCGGTTCAGCGTACAggc                                                                                                                                                                                                                                     >  1:391552/1‑300 (MQ=255)`  
                                                                           `gtACATTGAGGAATACTTAGGTGACTTACGGTCCCTTGAAAATCTCCAAGAGGCTATCGTCAAGATGTCCATGATTAGCTCTAAGGTTATCGGCTTAGTGAATCCTGCTGGTATCACCCAGCCACGCCGACTGACCAAAGCTCAGACTGCTGACTTCGTTACTGGTCGTCCAGAAGACATCTCGTTCCTCCAACTGGAGAAGCAAGCAGACTTTACTGTAGCTAACGCTGTAAGTGACGCTATCGAGGCGCGCCTTTCGTTTGCCTTTATGTTGAACTCTGCGGTTCAGCGTACAGGTGaa                                                                                                                                                                                                                                  <  1:33880/301‑1 (MQ=255)`  
                                                                            `tACATTGAGGAATACTTAGGTGACTTACGGTCCCTTGAAAATCTCCAAGAGGCTATCGTCAAGATGTCCATGATTAGCTCTAAGGTTATCGGCTTAGTGAATCCTGCTGGTATCACCCAGCCACGCCGACTGACCAAAGCTCAGACTGGTGACTTCGTTACTGGTCGTCCAGAAGACATCTCGTTCCTCCAACTGGAGAAGCAAGCAGACTTTACTGTAGCTAACGCCGTAAGTGACGCTATCGAGGCTCGCCTTTCGTTTGCCTTTATGTTGAACTCTGCGGTTCAGCGTACAGGTGAAc                                                                                                                                                                                                                                 <  1:81834/301‑1 (MQ=255)`  
                                                                               `caggAGCAATGCTTAGGTGACTTACGGTCCCTTGAAAATCTCCAAGAGGCTATCTTCAAGATGTCCATGATTAGCTCTAAGGTTATCGGCTTAGTGAATCCTGCTGGTATCACCCAGCCACGCCGACTGACCAAAGCTCAGACTGGTGACTTCGTTACTTGTCGTCCAGAGGACATCTCGTTCCTCCAACTGTAGAAGCAAGCAGACTTTACTGTAGCTAGCGCCGTAAGTGACGCTATCGAGGCTCGCCTTTCGTTTGCCTTTATGTTGAACCCTGCGGTTCAGCGTACAGGTGAACgtg                                                                                                                                                                                                                              <  1:539058/298‑1 (MQ=255)`  
                                                                                   `aGGAATACTTAGGTGACTTACGGTCCATTGAAAATCTCCAAGAGGCTATCGTCAAGATGTCCATGATTAGATCTAAGGTTATCGGCTTAGTGAATCCTGCTGGTATCACCCAGCCACGCCGACTGACCAAAGCTCAGACTGGTGACTTCGTTACTGGTCGTCCAGAAGACATCTCGTTCCTCCAACTGGAGAAGCAAGCAGACTTTACTGTAGCTAACGCCGTAAGTGACGCTATCGCGGCTCGCATTTCGTTTGCCTTTATGTTGAACTCTGCGGTTCAGCGTACAGGTGAACGTGTGAc                                                                                                                                                                                                                          <  1:25391/301‑1 (MQ=255)`  
                                                                                       `aTACTTAGGTGACTTACGGTCCCTTGAAAATCTCCAAGAGGCTATCGTCAAGATGTCCATGATTAGCTCTAAGGTTATCGGCTTAGTGAATCCTGCTGGTATCACCCAGCCACGCCGACTGACCAAAGCTCAGACTGGTGACTTCGTTACTGGTCGTCCAGAAGACATCTCGTTCCTCCAACTGGAGAAGCACGCAGACTTTACTTTATCTAACGCCGTAAGTGACGCTATCGCGGCTCGCCTTTCGTTTGCCTTTATGTTGAACTCTGCGGTTCAGCGTACAGGTGAACGTGTGAccgcc                                                                                                                                                                                                                      >  1:630596/1‑301 (MQ=255)`  
                                                                                       `aTACTTAGGTGACTTACGGTCCCTTGAAAATCTCCAAGAGGCTATCGTCAAGATGTCCATGATTAGCTCTAAGGTTATCGGCTTAGTGAATCCTGCTGGTATCACCCAGCCACGCCGACTGACCAAAGCTCAGACTGGTGACTTCGTTACTGGTCGTCCAGAAGACATCTCGTTCCTCCAACTGGAGAAGCAAGCAGACTTTACTGTAGCTAACGCCGTAAGTGACGCTATCGAGGCTCTCCTTTCGTTTGCCTTTATGTTGAACTCTGCGGTTCAGCGTActgtctcttatacacatctc                                                                                                                                                                                                                      >  1:522102/1‑282 (MQ=255)`  
                                                                                          `cTTAGGTGACTTACGGTCCCTTGAAAATCTCCAAGAGGCTATCGTCAAGATGTCCATGATTAGCTCTAAGGTTATCGGCTTAGTGAATCCTGCTGGTATCACCCAGCCACGCCGACTGACCAAAGCTCAGACTGGTGACTTCGTTACTGGTCGTCCAAAAGACATCTCGTTCCTCCAACTGGAGAAGCAAGCAGACTTTACTGTAGCTAACGCCGTAAGTGACGCTATCGAGGCTCGCCTTTCGTTTGCCTTTATGTTGAACTCTTCGGTTCAGCGTACAGGTGAACGTGTGAccgccgca                                                                                                                                                                                                                   >  1:346310/1‑299 (MQ=255)`  
                                                                                                 `gACTTACGGTCCCTTGAAAATCTCCAAGAGGCTATCGTCAAGATGTCCATGATTAGCTCTAAGGTTATCGGCTTAGTGAATCCTGCTGGTATCACCCAGCCACGCCGACTGACCAAAGCTCAGACTGGTGACTTCGTTACTGGTCGTCCAGAAGACATCTCGTTCCTCCAACTGGAGAAGCAAGCAGACTTTACTGTAGCTAACGCCGTAAGTGACGCTATCGAGGCTCGCCTTTCGTTTGCCTTTATGTTGAACTCTGCGGTTCAGCGTACAGGTGAACGTGTGACCGCCGAAGAGATTc                                                                                                                                                                                                            >  1:121727/1‑301 (MQ=255)`  
                                                                                                  `aCTTACGGTCCCTTGAAAATCTCCAAGAGGCTATCGTCAAGATGTCCATGATTAGCTCTAAGGTTATCGGCTTAGTGAATCCTGCTGGTATCACCCAGCCACGCCGACTGACCAAAGCTCAGACTGGTGACTTCGTTACTGGTCGTCCAGAAGACATCTCGTTCCTCCAACTGGAGAAGCAAGCAGACTTTACTGTAGCTAACGCCGTAAGTGACGCTATCGAGTCTCGCCTTTCGTTTGCCTTTATTTTGAACTTTGCGGTTCAGCGTACAGGTGAACGTGTGACCGCCGAATAGCtttg                                                                                                                                                                                                           >  1:461051/1‑299 (MQ=255)`  
                                                                                                      `ccGGTCCCTTGAAAATCTCCAAGAGGCTATCGTCAAGATGTCCATGATTCGCTCTAAGGTTATCGGCTTAGTGAATCCTGCTGGTATCACCCAGCCACGCCGACTGACCAAAGCTCAGACTGGTGACTTCGTTACTGGTCGTCCAGAAGACATCTCGTTCCTCCAACTGGAGAAGCAAGCAGACTTTACTGTAGCTAACGCCGTAAGTGACGCTATCGCGGCTCGCCTTTCGTTTGCCTTTATGTTGAACTCTGCGGTTCAGCGTACAGGTGAACGTGTGACCGCCGAAGAGATTCAGTAt                                                                                                                                                                                                       <  1:353537/300‑1 (MQ=255)`  
                                                                                                        `ggacccTTGAAAATCTCCAAGAGGCTATCGTCAAGATGTCCATGATTAGCTATAAGGTTATCGGCTTAGTGAATCCTGCTGGTATCACCCAGCCACGCCGACTGACCAAAGCTCAGACTGGTGACTTCGTTACTGGTCGTCCAGAAGACAACTCGTTCCTCCAACTGGAGAAGCAAGCAGACTTTACTGTAGCTAACGCCGTAAGTGACGCTATCGAGGCTCGCCTTTCGTTTGCCTTTATGTTAAACTCTGCGGTTCAGCGTACAGGTGAACGTGTGACCGCCGAAGAGATTCGGTATGt                                                                                                                                                                                                     <  1:373406/298‑1 (MQ=255)`  
                                                                                                           `cccTTGAAAATCTCCAAGAGGCTATCGTCAAGATGTCCATGATTAGCTCTAAGGTTATCGGCTTAGTGAATCCTGCTGGTATCACCCAGCCACGCCGACTGACCAAAGCTCAGACTGGTGACTTCGTTACTGGTCGTCCAGAAGACATCTCGTTCCTCCAACTGGAGAAGCAAGCAGACTTTACTGTAGCTAACGCCGTAAGTGACGCTATCGCGGCTCGCCTTTCGTTTGCCTTTATGTTGAACTCTGCGGTTCAGCGTACAGGTGAACGTGTGACCGCCGAAGAGATTCGGTATGTAGc                                                                                                                                                                                                  <  1:588416/301‑1 (MQ=255)`  
                                                                                                           `cccTTGAAAATCTCCAAGAGGCTATCGTCAAGATGTCCATGATTAGCTCTAAGGTTATCGGCTTAGTGAATCCTGCTGGTATCACCCAGCCACGCCGACTGACCAAAGCTCAGACTGGTGACTTCGTTACTGGTCGTCCAGAAGACATCTCGTTCCTCCAACTGGAGAAGCAAGCAGACTTTACTGTAGCTAACGCCGTAAGTGACGCTATCGCGGCCCGCCTTTCGTTTGCCTTTATGTTGAACTCTCCGGTTCAGCGTACAGGTGAACGTGTGACCGCCGAAAGGATTCGGTATGTAgt                                                                                                                                                                                                  >  1:135610/1‑300 (MQ=255)`  
                                                                                                              `ttGAAAATCTCCAAGAGGCTATCGTCAAGATGTCCATTATTAGCTCTAGGGTTATCGGCTTAGTGAATCCTGCTGGTATCACCCAGCCACGCCGACTGACCAAAGCTCAGACTGGTGACTTCGTTACTGGTCGTCCAGAAGCCATCTCGTTCCTCCAACTGGAGAAGCAAGCAGACTTCACTGTAGCTAACGCCGTAAGTGACTCTATCGAGTCTCGCCTTTCGTTTGCCTTTATGTTGAACTCTGCGGTTCAGCGTACAGGTGAACGTGTGACCGCCGAAGAGATTCGGTATGTAGCTTc                                                                                                                                                                                               <  1:599191/301‑1 (MQ=255)`  
                                                                                                                  `aaaTCTCCAAGAGGCTATTGTCAAGATGTCCATGATTAGCTCTAAGGTTATCGGCTTAGTGAATCCTGCTGGTATCACCCAGCCACGCCGACTGACCAAAGCTCAGACTGGTGACTTCGTTACTGGTCGTCCAGAAGACATCTCGTTCCTCCAACTGGAGAAGCAAGCAGACTTTACTGTAGCTAAAGCCGTAAGTGACGCTATCGAGGCTCGCCTTTCGTTTGCCTTTATGTTGAACTCTGCGGTTCAGCGTACAGGTGAACGTGTGACCGCCGAAGAGATTCGGTATGTAGCTTCTGaa                                                                                                                                                                                           <  1:16869/301‑1 (MQ=255)`  
                                                                                                                  `aaaTCTCCAAGAGGCTATCGTCAAGATGTCCATGATTAGCTCTAAGGTTATCGGCTTAGTGAATCCTGCTGGTATCACCCAGCCACGCCGACTGACCAAAGCTCAGACTGGTGACTTCGTTACTGGTCGTCCAGAAGACATCTCGTTCCTCCAACTGGAGAAGCAAGCAGACTTTACTGTAGCTAACGCCGTAAGTGACGCTATCGCGGCTCGCCTTTCGTTTGCCTTTATGTTGAACTCTGCGGTTCAGCGTACAGGTGAACGTGTGACCGCCGAAGAGATTCGGTATGTAGCTTCTGaa                                                                                                                                                                                           <  1:593894/301‑1 (MQ=255)`  
                                                                                                                     `tctcCAAGAGGCTATCGTCAAGATGTCCATGATTAGCTCTAAGGTTATCGGCTTAGTGAATCCTGCTGGTATCACCCAGCCACGCCGACTGACCAAAGCTCAGACTGGTGACTTCGTTACTGGTCGTCCAGAAGACATCTCGTTCCTCCAACTGGCGAAGCAAGCAGACTTTACTGTAGCTAACGCCGTAATTGACGATATCGCGGCTCGACTTTCGTTTGCCTTTATTTTTAACTCTGCGGGTCAGCGTACGGGTGCACGTGTGACCGCCGAATAGATTCGGTATGTAGCTTCGCAACtt                                                                                                                                                                                        >  1:618528/1‑301 (MQ=255)`  
                                                                                                                             `gacagttCGTCAAGATGTCCATTATTAGCTCGAAGGTTATCGGCTTAGTGAATCCTGCTGGTATCACCCAGCCACGCCGACTGACCAAAGCTCAGACTGGTGACTTCGTTACTGGTCGTCCAGAAGACATCTCGTTCCTCCAACTGGAGAAGCAAGCAGACTTTACTGTAGCTAACGCCGTAAGTGACGCTATCGCGGCTCGCCTTTCGTTTGCCTTTATGTTGAACTCTGCGGTTCAGCGTACAGGTGAACGTGTGACCGCCGAAGAGATTCGGTATGTAGCTTCTGAACTTGAAGATAc                                                                                                                                                                                <  1:331816/295‑1 (MQ=255)`  
                                                                                                                              `ggCTATCGTCAAGATGTCCATGATTAGCTCTAAGGTTATCGGCTTAGTGAATCCTGCTGGTATCACCCAGCCACGCCGACTGACCAAAGCTCAGACTGGTGACTTCGTTACTGGTCGTCCAGAAGACATCTCGTTCCTCCAACTGGAGAAGCAAGCAGACTTTACTGTAGCTAACGCCGTAAGTGACGCTATCGCGGCTCGCCTTTCGTTTGCCTTTATGTTGAACTCTGCGGTTCAGCGTACAGGTGAACGTGTGACCGCCGAAGAGATTCGGTATGTAGCTTCTGAACTTGAAGAtctt                                                                                                                                                                               >  1:248248/1‑298 (MQ=255)`  
                                                                                                                                `agaTCGTCAAGATGTCCATGATTAGCTCTAAGGTTATCGGCTTAGTGAATCCTGCTGGTATCACCCAGCCACGCCGACTGACCAAAGCTCAGACTGGTGACTTCGTTACTGGTCGTCCAGAAGACATCTCGTTCCTCCAACTGGAGAAGCAAGCAGACTTTACTGTAGCTAACGCCGTAAGTGACGCTATCGCGGCTCGCCTTTCGTTTGCCTTTATGTTGAACTCTGCGGTTCAGCGTACAGGTGAACGTGTGACCGCCGAAGAGATTCGGTATGTAGCTTCTGAACTTGAAGATACttt                                                                                                                                                                             <  1:566819/299‑1 (MQ=255)`  
                                                                                                                                  `aTCGTCAAGATGTCCATGATTAGCTCTAAGGTTATCGGCTTAGTGAATCCTGCTGGTATCACCCAGCCACGCCGACTGACCAAAGCTCAGACTGGTGACTTCGTTACTGGTCGTCCAGAAGACATCTCGTTCCTCCAACTGGAGAAGCAAGCCGACTTTACTGTAGCTAACGCCGTAAGTGACGCTATCGCGGCTCGCCTTTCGTTTGCCTTTATGTTGAACTCTGCGTTTCAGCGTACAGGTGAACGTGTGACCGCCGAAGAGATTCGGTATGTAGCTCCTGACCTTTAAGATACTTTAg                                                                                                                                                                           >  1:24158/1‑301 (MQ=255)`  
                                                                                                                                    `cGTCAAGATGTCCATGATTAGCTCTAAGGTTATCGGCTTAGTGAATCCTGCTGGTATCACCCAGCCACGCCGACTGACCAAAGCTCAGACTGGTGACTTCGTTACTGGTCGTCCAGAAGACATCTCGTTCCTCCAACTGGAGAAGCAAGCAGACTTTACTGTAGCTAACGCCGTAAGTGACGCTATCGAGGCTCGCCTTTCGTTTGCCTTTATGTTGAACTCTGCGGTTCAGCGTACAGGTGAACGTGTGACCGCCGAAGAGATTCGGTATGTAGCTTCTGAACTTGAAGATACTTTAggt                                                                                                                                                                         <  1:4897/301‑1 (MQ=255)`  
                                                                                                                                    `aagagacaggTCCATGATTAGCTCTAAGGTTATCGGCTTAGTGAATCCTGCTGGTATCACCCAGCCACGCCGACTGACCAAAGCTCAGACTGGTGACTTCGTTACTGGTCGTCCAGAAGACATCTCGTTCCTCCAACTGGAGAAGCAAGCAGACTTTACTGTAGCTAACGCCGTAAGTGACGCTATCGCGGCTCGCCTTTCGTTTGCCTTTATGTTGAACTCTGCGGTTCAGCGTACAGGTGAACGTGTGACCGCCGAAGAGATTCGGTATGTAGCTTCTGAACTTGAAGATACTTTAggt                                                                                                                                                                         <  1:44506/292‑1 (MQ=255)`  
                                                                                                                                      `tCAAGATGTCCATGATTAGCTCTAAGGTTATCGGCTTAGTGAATCCTGCTGGTATCACCCAGCCACGCCGACTGACCAAAGCTCAGACTGGTGACTTCGTTACTTGTCGTCCAGAAGACATCTCGTTCCTCCAACTGGAGAAGCAAGCAGACTTTACTGTAGCTAACGCCGTAAGTGACGCTATCGCGGCTCGCCTTTCGTTTGCCTTTATGTTGAACTCTGCGGTTCAGCGTACAGGTGAACGTGTGACCGCCGAAGAGATTCGGTATGTAGCTTCTGAACTTGAAGATACTTTAggtgg                                                                                                                                                                       <  1:645040/301‑1 (MQ=255)`  
                                                                                                                                          `aaaggccAGGATTAGCTGTAAGGTTATCGGCTTAGTTGATCCTGCTGGGATCACCCAGCCACGCCGACTGACCAAAGCTCAGACTGGTGACTGCGTTACTGGTCGTCCAGAAGACATCTCGTTCCTCCAACTGGAGAAGCAAGCAGACTTTACTGTAGCTAAAGCCGTAAGTGACGCTATCGAGGCTCGCCTTTCGTTTGCCTTTATGTTGAACTCTGCGTTTCAGCGTACAGGTGAACGTGTGACCGCCGAAGAGATTCGGTATGTAGCTTCTGAACTTGAAGATACTTTAGGTGG‑TGTc                                                                                                                                                                  <  1:500307/296‑1 (MQ=255)`  
                                                                                                                                                `agagacaggCTCTAAGGTTATCGGCTTAGTGAATCCTGCTGGTATCACCCAGCCACGCCGACTGACCAAAGCTCAGAATGGTGACTTCGTTACTGGTCGTCCAGAAGACATCTCGTTCCTCCAACTGGAGAAGCAAGCAGACTTTACTGTAGCTAACGCCGTAAGTGACGCTATCGCGGCTCGCCTTTCGTTTGCCTTTATGTTGAACTCTGCGGTTCAGCGTACAGGTGAACGTGTGACCGCCGAAGAGATTCGGTATGTAGCTTCTGAACTTGAAGATACTTTAGGTGG‑TGTCTActct                                                                                                                                                            <  1:298933/293‑1 (MQ=255)`  
                                                                                                                                                  `tGATTAGCTCTAAGGTTATCGGCTTAGTGAATCCTGCTGGTATCACCCAGCCACGCCGACTGACCAAAGCTCAGACTGGTGACTTCGTTACTGGTCGTCCAGAAGACATCTCGTTCCTCCAACTGGAGAAGCAAGCAGACTTTACTGTAGCTAACGCCGTAAGTGACGCTCTCGCGGCTCGCCTTTCGTTTGCCTTTATGTTGAACTCTGCGGTTCAGCGTACAGGTGAACGTGTGACCGCCGAAGAGATTCGGTATGTAGGTTCGTCACTTGAAGATACTTTAGGTGG‑TGTCTACTCTAt                                                                                                                                                          >  1:561389/1‑301 (MQ=255)`  
                                                                                                                                                   `gATTAGCTCTAAGGTTATCGGCTTAGTGAATCCTGCTGGTATCACCCAGCCACGCCGACTGACCAAAGCTCAGACTGGTGACTTCGTTACTGGTCGTCCAGAAGACATCTCGTTCCTCCAACTGGAGAAGCAAGCAGACTTTACTGTAGCTAACGCCGTAAGTGACGCTATCGCGGCTCGCCTTTCGTTTGCCTTTATGTTGAACTCTGCGGTTCAGCGTACAGGTGAACGTGTGACCGCCGAAGAGATTCGGTATGTAGCTTCTGAACTTGAAGATACTTTAGGTGG‑TGTCTActcctgt                                                                                                                                                         >  1:432355/1‑297 (MQ=255)`  
                                                                                                                                                    `tttAGCTCTAAGGTTATCGGCTTAGTGAATCCTGCTGGTATCACCCAGCCACGCCGACTGACCAAAGCTCAGACTGGTGACTTCGTTACTGGTCGTCCAGAAGACATCTCGTTCCTCCAACTGGAGAAGCAAGCAGACTTTACTGTAGCTAACGCCGTAAGTGACGCTATCGCGGCTCGCCTTTCGTTTGCCTTTATGTTGAACTCTGCGGTTCAGCGTACAGGTGAACGTGTGACCGCCGAAGAGATTCGGTATGTAGCTTCTGAACTTGAAGATACTTTAGGTGG‑TGTCTACTCTATcc                                                                                                                                                        <  1:162905/300‑1 (MQ=255)`  
                                                                                                                                                    `aTTAGCTCTAAGGTAATCGGCTTAGTGAATCCTGCTGGTATCACCAAGCCACGCCGACTGACCAAAGCTCAGACTGGTGACTTCGTTACTGGTCGTCCAGAAGACATCTCGTTCCTCCAACTGGAGAAGCAAGCAGACTTTACTGTAGCTAACGCCGTAAGTGACGCTATCGCGGCTCGCCTTTCGTTTGCCTTTATGTTGAACTCTGCGGTTCAGCGTACAGGTGAACGTGTGACCGCCGAAGAGATTCGGTATGTAGCTTCTGAACTTGAAGATACTTTAGGTGG‑TGTCTACTCTATcc                                                                                                                                                        <  1:192252/301‑1 (MQ=255)`  
                                                                                                                                                        `gCTCTAAGGTTATCGGCTTAGTGAATCCTGCTGGTATCACCCAGCCACGCCGACTGACCAAAGCTCAGACTGGTGCCTTCGTTACTGGTCTTCCAGAAGACATCTCGTTCCTCCAACTGGAGAAGCAAGCAGACTTTACTGTAGCTAACGCCGTAAGTGACGCTATCGAGGCTCGCCTTTCGTTTGCCTTTATGTTGAACTCTGCGGTCAAGCGTAAAGGTGAACGTTTGACCGCCGAAGAGATTCGGTATGTAGCTTCTGAACTTGAAGATACTTTAGGTGG‑TGTCTACTCTATCCcttt                                                                                                                                                    >  1:100141/1‑300 (MQ=255)`  
                                                                                                                                                        `gCTCTAAGGTTATCGGCTTAGTGAATCCTGCTGGTATCACCCAGCCACGCCGACTGACCAAAGCTCAGACTGGTGACTTCGTTACTGGTCGTCCAGAAGACATCTCGTTCCTCCAACTGGAGAAGCAAGCAGACTTTACTGTAGCTAACGCCGTAAGTGACGCTATCGCGGCTCGCCTTTCGCTTGCCTTTATGTTGAACTCTGCGGTTCAGCGTACAGGTGAACGTGTGACCGCCGAAGAGATTCGGTATGTAGCTTCTGAACTTGAAGATACTTTAGTTGG‑TGTCTACTCTATCCtttt                                                                                                                                                    >  1:326334/1‑300 (MQ=255)`  
                                                                                                                                                         `gatgtgtataagagacagaGTGAATCCTGCTGGTATCACCCAGCCACGCCGACTGACCAAAGCTCAGACTGGTGACTTCGTTACTGGTCGTCCAGAAGACATCTCGTTCCTCCAACTGGAGAAGCAAGCAGACTTTACTGTAGCTAAAGCCGTAAGTGACGCTATCGAGGCTCGCCTTTCGTTTGCCTTTATGTTGAACTCTGCGGTTCAGCGTACAGGTGAACGTGTGACCGCCGAAGAGATTCGGTATGTAGCTTCTGAACTTGAAGATACTTTAGGTGG‑TGTCTACTCTATCCTTtct                                                                                                                                                   <  1:150724/283‑1 (MQ=255)`  
                                                                                                                                                               `ggTTATCGGCTTAGTGAATCCTGCTGGTATCTCCCAGCCACGCCGACTGACCAAAGCTCAGACTGGTGACTTCGTTACTGGTCGTCCAGAAGACATCTCGTTCCTCCAACTGGAGAAGCAAGCAGACTTTACTGTAGCTAACGCCGTAAGTGACGCTATCGCGGCTCGCCTTTCGTTTGCCTTTATGTTGAACTCTGCGGTTCAGCGTACAGGTGAACGTGTGACCGCCGAAGAGATTCGGTATGTAGCTTCTGAACTTGAAGATACTTTAGGTGGCTGTCtcttatacacatctcctatc                                                                                                                                              >  1:197403/1‑282 (MQ=255)`  
                                                                                                                                                                `gTTATCGGCTTAGTGAATCCTGCTGGTATCACCCAGCCACGCCGACTGACCAAAGCTCAGACTGGTGACTTCGTTACTGGTCGTCCAGAAGACATCTCGTTCCTCCAACTGGAGAAGCAAGCAGACTTTACTGTAGCTAACGCCGTAAGTGACGCTATCGCGGCTCGCCTTTCGTTTGCCTTTATGTTGAACTCTGCGGTTCAGCGTACAGGTGAACGTGTGACCGCCGAAGAGATTCGGTATGTAGCTTCTGAACTTGAAGATACTTTAGGTGG‑TGTCTACTCTATCCTTTCTCAAGAAt                                                                                                                                            >  1:532516/1‑301 (MQ=255)`  
                                                                                                                                                                `gTTATCGGCTTAGTGAATCCTGCTGGTATCACCCAGCCACGCCGACTGACCAAAGCTCAGACTGGTGACTTCGTTACTGGTCGTCCAGAAGACATCTCGTTCCTCCAACTGGAGAAGCAAGCAGACTTTACTGTAGCTAACGCCGTAAGTGACGCTATCGAGGCTCGCCTTTCGTTTGCCTTTATGTTGAACTCTGCGGTTCAGCGTACAGGTGAACGTGTGACCGCCGAAGAGATTCGGTATGTAGCTTCTGAACTTGAAGATACTTTAGGTGG‑TGTCTACTCTATCCTTTCTCAAGAAt                                                                                                                                            >  1:222239/1‑301 (MQ=255)`  
                                                                                                                                                                     `agaaagatgcaaaccTGCTGGTATCACCCAGCCACGCCGACTGACCAAAGCTCAGACTGGTGACTTCGTTACTGGTCGTCCAGAAGACATCTCGTTCCTCCAACTGGAGAAGCAAGCAGACTGTACTGTAGCTAACGCCGTAAGTGACGCTATCGAGGCTCGCCTTTCGTTTGCCTTTATGTTGAACTCTGCGGTTCAGCGTACAGGTGAACGTGTGACCGCCGAAGAGATTCGGTATGTAGCTTCTGAACTTGAAGATACTTTAGGTGG‑TGTCTACTCTATCCTTTCTCAAGAATTACaa                                                                                                                                       <  1:426872/288‑1 (MQ=255)`  
                                                                                                                                                                           `agtaaacacgtctgGTATCACCCAGGCATGTCGACTGACCAAAGCTCAGACTGGTGACTTCGTTACTGATCGTCCAGAAGACAGCTCGTTCCTTCAACTGGAGAAGCAAGCAGACTTTACTGTAGCTAACGGCGTAAGTGACGCTATCGAGGTTCGCTTTTCGCTTGCCTTTATGTTGAACTCTGCGGTTCAGCGTACAGGTGAACGTGTGACCGCCGAAGAGATTCGGTATGTAGCTTCTGTACTTGAAGCTACTTTGCGTGG‑TTTGTACTCTATCCTTTCTCAAGAATTACAGTTGCct                                                                                                                                 <  1:286125/290‑1 (MQ=255)`  
                                                                                                                                                                           `aGTGAATCCTGCTGGTATCACCCAGCCACGCCGACTGACCAAAGCTCAGACTGGTGACTTCGTTACTGGTCGTCCAGAAGACATCTCGTTCCTCCAACTGGAGAAGCAAGCAGACTTTACTGTATCTAACGCCGTAAGTGACGCTATCGCGGCTCGCCTTTCGTTTGCCTTTATGTTGAACTCTGCGGTTCAGCGTACAGGTGAACGTGTGACCGCCGTAGAGCTTCGGTATGTAGCTTCTGAACTTGAAGATAATTTAGGTGG‑TGTCTACTCTATCCTTtctctgtctcttatacacatc                                                                                                                                 >  1:141298/1‑284 (MQ=255)`  
                                                                                                                                                                           `aGTGAATCCTGCTGGTATCACCCAGCCACGCCGACTGACCAAAGCTCAGACTGGTGACTTCGTTACTGGTCGTCCAGAAGACATCTCGTTCCTCCAACTGGAGAAGCAAGCAGACTTTACTGTAGCTAACGCCGTAAGTGACGCTATCGAGGCTCGCCTTTCGTTTGCCTTTATGTTGAACTCTGCGGTTCAGCGTACAGGTGAACGTGTGACCGCCGAAGAGCTTCGGTATGTAGCTTCTGAACTTGAAGATACTTTAGGTGG‑TGGCTACTCTATCCTTtctgtctcttatacccatctc                                                                                                                                 >  1:650197/1‑283 (MQ=255)`  
                                                                                                                                                                                `aTCCTGCTGGTATCACCCAGCCACGCCGACTGACCAAAGCTCAGACTGGTGACTTCGTTACTGGTCGTCCAGAAGACATCTCGTTCCTCCAACTGGAGAAGCAAGCAGACTTTACTGTAGCTAACGCCGTAAGTGACGCTATCGCGGCTCGCCTTTCGTTTGCCTTTATGTTGAACTCTGCGGTTCAGCGTACAGGTGAACGTGTGACCGCCGAAGAGATTCGGTATGTAGCTTCTGAACTTGAAGATACTTTAGGTGG‑TGTCTACTCTATCCTTTCTCAAGAATTACAATTGCCTCTGGt                                                                                                                            >  1:49496/1‑301 (MQ=255)`  
                                                                                                                                                                                `aTCCTGCTGGTATCACCCAGCCACGCCGACTGACCAAAGCTCAGACTGGTGACTTCGTTACTGGTCGTCCAGAAGACATCTCGTTCCTCCAACTGGAGAAGCAAGCAGACTTTACTGTAGCTAACGCCGTAAGTGACGCTATCGAGGCTCGCCTTTCGTTTGCCTTTATGTTGAACTCTGCGGTTCAGCGTACAGGTGAACGTGTGACCGCCGAAGCGATTCGGTATGTAGCTTCTGAACTTGAAGATACTTTAGGTGG‑TGTCTACTCTATCCTTTCTCAAGAATTACAATTGCCTCTGGt                                                                                                                            >  1:45556/1‑301 (MQ=255)`  
                                                                                                                                                                                       `tgGTATCACCCAGCCACGCCGACTGACCAAAGCTCAGACTGGTGACTTCGTTACTGGTCGTACAGAAGACATCTCGTTCCTCCAACTGGAGAAGCAAGCAGACTTTACTGTAGCTAACGCCGTAAGTGACGCTATCGCGGCTCGCCTTTCGTTTGCCTTTATGTTGAACTCTGCGGTTCAGCGTACAGGTGAACGTGTGACCGCCGAAGAGATTCGGTATGTAGCTTCTGAACTTGAAGATACTTTAGGTGG‑TGTCTACTCTATCCTTTCTCAAGAATTACAATTGCCTCTGGTACGAGTg                                                                                                                     <  1:180562/301‑1 (MQ=255)`  
                                                                                                                                                                                        `ggTATCACCCAGCCACGCCGACTGACCAAAGCTCAGACTGGTGACTTCGTTACTGGTCGTCCAGAAGACATCTCGTTCCTCCAACTGGAGAAGCAAGCAGACTTTACTGTAGCTAACGCCGTAAGTGACGCTATCGAGGCTCGCCTTTCGTTTGCCTTTATGTTGAACTCTGCGTTTCAGCGTACAGGTGAACGTGTGACCGCCGAAGAGATTCGGTATGTAGCTTCTGAACTTGAAGATACTTTAGGTGG‑TGTCTACTCTATCCTTTCTCAAGAATTACAATTGCCTCTggctgtctctt                                                                                                                    >  1:612306/1‑292 (MQ=255)`  
                                                                                                                                                                                         `gTATCACCCAGCCACGCCGACTGACCAAAGCTCAGACTGGTGACTTCGTTACTGGTCGTCCAGAAGACATCTCGTTCCTCCAACTGGAGAAGCAAGCAGACTTTACTGTAGCTAACGCCGTAAGTGACGCTATCGCGGCTCGCCTTTCGTTTGCCTTTATGTTGAACTCTGCGGTTCAGCGTACAGGTGAACGTGTGACCGCCGAAGAGATGCGGTATGTAGCTTCTGAACTTGAAGATACTTTAGGTGG‑TGTCTACTCTATCCTTTCTCAAGAATTACAATTGCCTCTGGTACGAGTG‑ct                                                                                                                  >  1:367878/1‑301 (MQ=255)`  
                                                                                                                                                                                             `cACCCAGCCACGCCGACTGACCAAAGCTCAGACTGGTGACTTCGTTACTGGTCGTCCAGAAGACATCTCGTTCCTCCAACTGGAGAAGCAAGCAGACTTTACTGTAGCTAACGCCATAAGTGACGCTATCGCGGCTCGCCTTTCGTTTGCCTTTATGTTGAACTCTGCGGTTCAGCGTACAGGTGAACGTGTGACCGCCGAAGAGATTCGGTATGTAGCTTCTGAACTTGAAGATACTTTAAGTGG‑TGTCTACTCTATCCTTTCTCAAGAATTACAATTGCCTCTGGTACGAGAG‑CTCttt                                                                                                              >  1:228098/1‑300 (MQ=255)`  
                                                                                                                                                                                                 `cAGCCACGCCGACTGACCAAAGCTCAGACTGGTGACTTCGTTACTGGTCGTCCAGAAGACATCTCGTTCCTCCAACTGGAGAAGCAAGCAGCCTTTACTGTAGCTAACGCCGTAAGTGACGCTATCGCGGCTCGCCTTTCGTTTGCCTTTATGTTGAACTCTGCGGTTCAGCGTACAGGTGAACGTGTGACCGCCGAAGAGATTCGGTATGTAGCTTCTGAACTTGAAGATCCTTTAGGTGG‑TGTCTACTCTATCCTTTCTCAAGAATTACAATTGCCTCTGGTACGAGTT‑CTCTTTAAGc                                                                                                          >  1:107902/1‑301 (MQ=255)`  
                                                                                                                                                                                                 `cAGCCACGCCGACTGACCAAAGCTCAGACTGGTGACTTCGTTACTGGTCGTCCAGAAGACATCTCGTTCCTCCAACTGGAGAAGCAAGCAGACTTTACTGTAGCTAAAGCCGTAAGTGACGCTATCGAGGCTCGCCTTTCGTTTGCCTTTATGTTGAACTCTGCGGTTCAGCGTACAGGTGAACGTGTGACCGCCGAAGAGATTCGGTATGTAGCTTCTGAACTTGAAGATACTTTAGGTGG‑TGTCTACTCTATCCTTTCTCAAGAATTACAATTGCCTCTGGTACGAGTG‑CTCTTGAAGc                                                                                                          >  1:385250/1‑301 (MQ=255)`  
                                                                                                                                                                                                       `tgtataagagacagaGCTCAGACTGGTGACTTCGTTACTGGTCGTCCAGAAGACATCTCGTTCCTCCAACTGGAGAAGCAAGCAGACTTTACTGTAGCTAACGCCGTAAGTGACGCTATCGAGGCTCGCCTTTCGTTTGCCTTTATGTTGAACTCTGCGGTTCAGCGTACAGGTGAACGTGTGACCGCCGAAGAGATTCGGTATGTAGCTTCTGAACTTGAAGATACTTTAGGTGG‑TGTCTACTCTATCCTTTCTCAAGAATTACAATTGCCTCTGGTACGAGTG‑CTCTTGAAGCAACTAc                                                                                                    <  1:414294/287‑1 (MQ=255)`  
                                                                                                                                                                                                           `gACTGACCAAAGCTCAGACTGGTGACTTCGTTACTGGTCGTCTAGAAGACATCTCGTTCCTCCAACTGGAGAAGCAAGCAGACTTTACTGTAGCTAACGCCGAAAGTGACGCTATCGCGGCTCGCCTTTCGTTTGCCTTTATGTTTAACTCTGCGGTTCAGCGTACAGGTGAACGTGTGACCGCCGAAGAGATTCGGTATGTAGCTTCTGAACTTGAAGATACTTTAGGTGG‑TGTCTACTCTATCCTTTCTCAAGAATTACAATTGCCTCTGGTACGACTGTCTCttatacacatctccga                                                                                                 >  1:505027/1‑287 (MQ=255)`  
                                                                                                                                                                                                            `aCTGACCAACGCTCAGACTGGTGACTTCGTTACTGGTCGTCCAGAAGACATCTCGTTCCTCCAACTGGAGAAGCAAGCAGACTTTACTGTAGCTAACGCCGTAAGTGACGCTATCGCGGCTCGCCTTTCGTTTGCCTTTATGTTGAACTCTGCGGTTCAGCGTACAGGTGAACGTGTTACCGCCGAAGAGATTCGGTATGTAGCTTCTGAACTTGAAGATACTTTAGGTGG‑TGTCTACTCTATCCTTTCTCAAGAATTACAATTGCCTCTGGTACGAGTG‑CTCTTGAAGCAACTACAAGcc                                                                                               <  1:402592/301‑1 (MQ=255)`  
                                                                                                                                                                                                                     `aaacaggACTGGTGACTTCGTTACTGGTCGTCCAGAAGACATCTCGTTCCTCCAACTGGAGAAGCAAGCAGACTTTACTGTAGCTAACGCCGTAAGTGACGCTATCGCGGCTCGCCTTTCGTTTGCCTTTATGTTGAACTCTGCGGTTCAGCGTACAGGTGAACGTGTGACCGCCGAAGAGATTCGGTATGTAGCTTCTGAACTTGAAGATACTTTAGGTGG‑TGTCTACTCTATCCTTTCTCAAGAATTACAATTGCCTCTGGTACGAGTG‑CTCTTGAAGCAACTACAAGCCACGCAACAg                                                                                      <  1:655303/295‑1 (MQ=255)`  
                                                                                                                                                                                                                     `aGCTCAGACTGGTGACTTCGTTACTGGTCGTCCAGAAGACATCTCGTTCCTCCAACTGGAGAAGCAAGCAGACTTTACTGTAGCTAACGCCGTAAGTGACGCTATCGCGGCTCGCCTTTCGTTTGCCTTTATGTTGAACTCTGCGGTTCAGCGTACAGGTGAACGTGTGACCGCCGAAGATATTCGGTATGTAGCTTCTGAACTTGAAGATACTTTAGGTGG‑TGTCTACTCTATCCTTTCTCAAGAATTACAATTGCCTCTGGTACGAGTG‑CTCTTGAAGCAACTACAAGCCACGCAACAg                                                                                      >  1:113606/1‑301 (MQ=255)`  
                                                                                                                                                                                                                     `aGCTCAGACTGGTGACTTCGTTACTGGTCGTCCAGAAGACATCTCGTTCCTCCAACGGGAGAAGCAAGCAGACTTTACTGTAGCTAACGCCGTAAGTGACGCTATCGCGGCTCGCCTTTCGTTTGCCTTTATGTTGAACTCTGCGGTTCAGCGTACAGGTGAACGTGTGACCGCCGAAGAGATTCGGGATGTAGCTTCTGAGCTTGAAGATACTTTAGGTGG‑TGTCTACTCTATCCTTTCTCAAGAATTACAATTGCCTCTGGTACGAGTG‑CTCTTGAAGCAACTACAAGCCACGCAACAg                                                                                      <  1:74434/301‑1 (MQ=255)`  
                                                                                                                                                                                                                      `gCTCAGACTGGTGACTTCGTTACTGGTCGTCCAGAAGACATCTCGTTCCTCCAACTGGAGAAGCAAGCAGACTTTACTGTAGCTAAAGCCGTAAGTGACGCTATCGAGGCTCGCCTTTCGTTTGCCTTTATGTTGAACTCTGCGGTTCAGCGTACAGGTGAACGTGTGACCGCCGAAGAGATTCGGTATGTAGCTTCTGAACTTGAAGATACTTTAGGTGG‑TGTCTACTCTATCCTTTCTCAAGAATTACAATTGCCTCTGGTACGAGTG‑CTCTTGAAGCAACTACAACCCACGCAACAGa                                                                                     >  1:360147/1‑301 (MQ=255)`  
                                                                                                                                                                                                                        `gagacagggTGACTTCGTTACTGGTCGTCCAGAAGACATCTCGTTCCTCCAACTGGAGAAGCAAGCAGACTTTACTGTAGCTAACGCCGTAAGTGACGCTATCGCGGCTCGCCTTTCGTTTGCCTTTATGTTGAACTCTGCGGTTCAGCGTACAGGTGAACGTGTGACCGCCGAAGAGATTCGGTATGTAGCTTCTGAACTTGAAGATACTTTAGGTGG‑TGTCTACTCTATCCTTTCTCAAGAATTACAATTGCCTCTGGTACGAGTG‑CTCTTGAAGCAACTACAAGCCACGCAACAGAtt                                                                                   <  1:582295/294‑1 (MQ=255)`  
                                                                                                                                                                                                                                      `tCGTTACTGGTCGTCCAGAAGACATCTCGTTCCTCCAACTGGAGAAGCAAGCAGACTTTACTGTAGCTAACGCCGTAAGTGACGCTATCGAGGCTCGCCTTTCGTTTGCCTTTATGTTGAACTCTGCGGTTCAGCGTACAGGTGAACGTGTGACCGCCGAAGAGATTCGGTATGTAGCTTCTGAACTTGAAGATACTTTAGGTGG‑TGTCTACTCTATCCTTTCTCAAGAATTACAATTGCCTCTGGTACGAGTG‑CTCTTGAAGCAACTACAAGCCACGCAACAGATTCCTGAGTTAccctg                                                                     >  1:511290/1‑298 (MQ=255)`  
                                                                                                                                                                                                                                       `cGTTACTGGTCGTCCAGAAGACATCTCGTTCCTCCAACTGGAGAAGCAAGCAGACTTTACTGTAGCTAACGCCGTAAGTGACGCTATCGAGGCTCGCCTTTCGTTTGCCTTTATGTTGAACTCTGCGGTTCAGCGTACAGGTGAACGTGTGACCGCCGAAGAGATTCGGTATGTAGCTTCTTAACTTGAAGATACTTTAGGTGG‑TGTCTACTCTATCCTTTCTCAAGAATTACAATTGCCTCTGGTACGAGTG‑CTCTTGAAGCAACTACAAGCCACGCAACAGATTCCTGAGTTACCTAAg                                                                    <  1:268378/301‑1 (MQ=255)`  
                                                                                                                                                                                                                                        `gTTACTGGTCGTCCAGAAGACATCTCGTTCCTCCAACTGGAGAAGCAAGCAGACTTTACTGTAGCTAACGCCGTAAGTGACGCTAGCGCGGCTCGCCTTTCGTTTGCCTTTATGTTGAACTCTGCGGTTCAGCGTACAGGTGAACGTGTGACCGCCGAAGAGATTCGGTATGTAGCTTCTGAACTTGAAGATACTTTAGGTGG‑TGTCTACTCTATCCTTTCTCAAGAATTACAATTGCCTCTGGTACGAGTG‑CTCTTGAAGCAACTACAAGCCACGCAACAGATTCCTGAGTTACCTAAgg                                                                   <  1:69437/301‑1 (MQ=255)`  
                                                                                                                                                                                                                                                  `gtcCAGAAGACATCTCGTTCCTCCAACTGGAGAAGCAAGCAGACTTTACTGTAGCTAACGCCGTAAGTGACGCTATCGCGGCTCGCCTTTCGTTTGCCTTTATGTTGAACTCTGCGGTTCAGCGTACAGGTGAACGTGTGACCGCCGAAGAGATTCGGTATGTAGCTTCTGAACTTGAAGATACTTTAGGTGG‑TGTCTACTCTATCCTTTCTCAAGAATTACAATTGCCTCTGGTACGAGTT‑CTCTTGAAGCAACTCCAAGCCACGCAACCGATTCCTGAGTTACCTAAGGAAGCCGTaga                                                         >  1:660261/1‑301 (MQ=255)`  
                                                                                                                                                                                                                                                      `cgcggagatgtgtataagagacagggAGAATCAAGCAGACTTTACTGTAGCTAACGCCGTAAGTGACGCTATCGAGGCTCGCCTTTCGTTTGCCTTTATGTTGAACTCTGCGGTTCAGCGTACAGGCGAACGTGTGACCGCCGAAGAGATTCGGTATGTAGCTTCTGAACTTGAAGATACTTTAGGTGG‑TGTCTACTCTATCCTTTCTCAAGAATTACAATTGCCTCTGGTACGAGTG‑CTCTTGAAGCAACTACAAGCCACGCAACAGATTCCTGAGTTACCTAAGGAAGCCGTAGAGCCa                                                     <  1:85990/277‑1 (MQ=255)`  
                                                                                                                                                                                                                                                             `aTCTCGTTCCTCCAACTGGAGAAGCAAGCAGACTTTACTGTAGCTAACGCCGTAAGTGACGCTATCGCGGCTCGCCTTTCGTTTGCCTTTATGTTGAACTCTGCGGTTCAGCGTACAGGTGAACGTGTGACCGCCGAAGAGATTCGGTATGTAGCTTCTGAACTTGAAGATACTTTAGGTGG‑TGTCTACTCTATCCTTTCTCAAGAATTACAATTGCCTCTGGTACGAGTG‑CTCTTGAAGCAACTACAAGCCACGCAACAGATTCCTGAGTTACCTAAGGactgtctcttatacacctctc                                              >  1:279121/1‑281 (MQ=255)`  
                                                                                                                                                                                                                                                                 `ggctcggagatgtgtataagagacaggACTTTACTGTAGCTAACGCCGTAAGTGACGCTATCGAGGCTCGCCTTTCGTTTGCCTTTATGTTGAACTCTGCGGTTCAGCGTACAGGTGAACGTGTGACCGCCGAAGAGATTCGGTATGTAGCTTCTGAACTTGAAGATACTTTAGGTGG‑TGTCTACTCTATCCTTTCTCAAGAATTACAATTGCCTCTGGTACGAGTG‑CTCTTGAAGCAACTACAAGCCACGCAACAGATTCCTGAGTTACCTAAGGAAGCCGTAGAGCCAACCATTAGTAc                                          <  1:556060/275‑1 (MQ=255)`  
                                                                                                                                                                                                                                                                 `cGTTCGTCCAACTGCAGATGCACGCAGACGTTACGGTATCTAACTCCGTAAGTGACGCTATCGCGTCTCGTCTTTCGTTTGCCTTTATGTTGAACTCTGCGGTTCAGCGTACAGGTGAACGTGTGACCGCCGAAGAGATTCGATATGTAGCTTCTGAACTAAAAGATACTGTATGTGG‑TGTCTTCTCTATCCTTTCTCAAGAATTACAATTGCCTCTGGTACGAGTT‑CTCTTAAAGCAACTACAAGCCACGCAACAGATTCCTGAGTTACCTATGTAAGCCGTAGAGCCAACCATTAGTAc                                          <  1:157493/301‑1 (MQ=255)`  
                                                                                                                                                                                                                                                                 `cGTTCCTCCAACTGGAGAAGCAAGCAGACTTTACTGTAGCTAACGCCGTAAGTGACGCTATCGCGGCTCGCCTTTCGTTTGCCTTTATGTTGAACTCTGCGGTTCAGCGTACAGGTGAACGTGTGACCGCCGAAGAGATTCGGTATGTAGCTTCTGAACTTGAAGATACTTTAGGTGG‑TGTCTACTCTATCCTTTCTCAAGAATTACAATTGCCTCTGGTACGAGTG‑CTCTTGAAGCAACTACAAGCCACGCAACAGATTCCTGAGTTACCTAAGGAAGCCGTAGAGCCAACCATTAGTAc                                          <  1:311507/301‑1 (MQ=255)`  
                                                                                                                                                                                                                                                                 `cGTTCCTCCAACTGGAGAAGCAAGCAGACTTGACGTTAGCTAACGCCGTAAGTGACGCTATCGCGGCTCGCCTTTCGTTTGCCTTTATGTTGAACTCTGCGGTTCAGCGTACAGGTGAACGTGTGACCGCCGAAGAGATTCGGTATGTAGCTTCTGAACTTGAAGATACTTTAGGTGG‑TGTCTACTCTATCCTTTCTCAAGAATTACAATTGCCTCTGGTACGAGTG‑CTCTTGAAGCAACTACAAGCCACGCAACAGATTCCTGAGTTACCTAAGGAAGCCGTAGAGCCAACCATTAGTAc                                          <  1:60654/301‑1 (MQ=255)`  
                                                                                                                                                                                                                                                                 `cGTTCCTACAACTGGAGAAGCAAGCAGACTTTACTGTAGCTAACGCCGTAAGTGACGCTATCGCTGCTCGCCTTACGTGTGCCCTTAAGTTGAGCTCTGCGGTTCAGCGTACAGGTGAACGTGTGACCGCCGAAGATATTCGGTATGTAGCTTCTGAACTTGAATATACTTTAGGTGG‑TGTCTACTCTATCCTTTCTCAAGAATTACAATTGCCTCTGGTACGAGTG‑CTCTTGAAACAACTACAAGCCACGCAACAGATTCATGAGTTACCTAAGGAAGCCGTAGAGCCAACCATTAGTAc                                          <  1:466512/301‑1 (MQ=255)`  
                                                                                                                                                                                                                                                                 `cGTTCCGCCAACGGGAGAAGAAAGCAGACTTTACGGTAGCTAACGCCGTAAGTGGCGCGAGCGCGGCTCGCCTTTCGTGTGCCGTTATGCTGAACGCTGCGGTGCAGCGTACAGGTGAACGTGTGACCGCCGAAGGGATTCGGTATGTAGCTTCTGAACATGAATATACTTTAGGTGG‑TGTCTACTCTATCCTTTCTCAAGAATAACGATTGCCTCTGGTACGAGTG‑CTCTTGAAGCAACTACAAGCCACGCAACAGATTCCTGAGTTACCTAAGGAAGCCGTAGAGCCAACCATTAGTAc                                          <  1:203362/301‑1 (MQ=255)`  
                                                                                                                                                                                                                                                                     `cctccAACTGGAGAAGCAAGCAGACTTTACTGTAGCTAACGCCGTAAGTGACGCTATCGCGGCTCGCCTTTCGTTTGCCTTTATGTTGAACTCTGCGGTTCAGCGTACAGGTGAACGTGTGACCGCCGAAGAGATTCGGTATGTATCTTCTGAACTTGAAGATACTTTAGGTGG‑TGTCTACTCTATCCTTTCTCAAGAATTACAATTGCCTCTGGTACGAGTG‑CTCTTGAAGCAACTACAAGCCACGCAACAGATTCCTGAGTTACCTAAGGAAGCCTTAGAGCCAACCATTAGTACAGGt                                      >  1:450019/1‑301 (MQ=255)`  
                                                                                                                                                                                                                                                                      `ctccAACTGGAGAAGCAAGCAGACTTTACTGTAGCTAACGCCGTAAGTGACGCTATCGAGGCTCGCCTTTCGTTTGCCTTTATGTTGAACTCTGCGGTTCAGCGTACAGGTGAACGTGTGACCGCCGAAGAGATTCGGTATGTAGCTTCTGAACTTGAAGATACTTTAGGTGG‑TGTCTACTCTATCCTTTCTCAAGAATTACAATTGCCTCTGGTACGAGTG‑CTCTTGAAGCAACTACAAGCCACGCAACAGATTCCTGAGTTACCTAAGGAAGCCGTAGAGCCAACCATTAGTACAGGTc                                     <  1:667403/301‑1 (MQ=255)`  
                                                                                                                                                                                                                                                                      `ctccAACTGGAGAAGCAAGCAGACGTTACGTTAGCTAACGCCGTAAGTGACGCTATCGAGGATCGCCTTTCGTTTGCCTTTATGTTGAACTCTGCGGTTCAGCGTACAGGTGAACGTGTGACCGCCGAAGAGATTCGGTATGTAGCTTCTGAACTTGAAGATACTTTAGGTGG‑TGTCTACTCTATCCTTTCTCAAGAATTACAATTGCCTCTGGTACGAGTG‑CTCTTGAAGCAACTACAAGCCACGCAACAGATTCCTGAGTTACCTAAGGAAGCCGTAGAGCCAACCATTAGTACAGGTc                                     <  1:635872/301‑1 (MQ=255)`  
                                                                                                                                                                                                                                                                        `ccAACTGGAGGAGCAAGCAGACTTTACTGTAGCTAACGCCGTAAGTGACGCTATCGAGGCTCGCCTTTCGTTTGCCTTTATGTTGAACTCTGCGGTTCAGCGTACAGGTGAACGTGTGACCGCCGAAGAGATTCGGTATGTAGCTTCTGAACTTGAAGATACTTTAGGTGG‑TGTCTACTCTATCCTTTCTCAAGAATTACAATTGCCTCTGGTACGAGTG‑CTCTTGAAGCAACTACAAGCCACGCAACAGATTCCTGAGTTACCTAAGGAAGCCGTAGAGCCAACCATTAGTACAGGTCTg                                   >  1:485095/1‑301 (MQ=255)`  
                                                                                                                                                                                                                                                                            `atgtgtataagagacagcTTTACTGTAGCTAACGCCGTAAGTGACGATATCGAGGCTCGCCTTTCGTTTGCCTTTATGTTGAACTCTGCGGTTCAGCGTACAGGTGAACGTGTGACCGCCGAAGAGATTCGGTATGTAGCTTCTGAACTTGAAGATACTTTAGGTGG‑TGTCTACTCTATCCTTTCTCAAGAATTACAATTGCCTCTGGTACGAGTG‑CTCTTGAAGCAACTACAAGCCACGCAACAGATTCCTGAGTTACCTAAGGAAGCCGTAGAGCCAACCATTAGTACAGGTCTGGAAg                               <  1:408819/284‑1 (MQ=255)`  
                                                                                                                                                                                                                                                                                   `agcaagtaGACTTTACTGTAGCTAAAGCCGTAAGTGACGCTATCGAGTCTCGCCTTTCGTTTGCCTTTATGTTGAACTCTGCGGTTCAGCGTACAGGTGAACGTGTGACCGCCGAAGAGATTCGGTATGTAGCTTCTGAACTTGAAGATACTTTAGGTGG‑TGTCTACTCTATCCTTTCTCAAGAATTACAATTGCCTCTGGTACGAGTG‑CTCTTGAAGCAACTACAAGCCACGCAACAGATTCCTGAGTTACCTAATGACGCCGTAGAGCCAACCATTAGTACAGGTCTGGATGCAATTgg                        >  1:478863/1‑301 (MQ=255)`  
                                                                                                                                                                                                                                                                                           `taCTTTACTGTAGCTAACGTCGTAAGTGACGCTATAGCGGCTCGCCTTTCGTTTGCCTTTATGTTGAACACTGCGGTTCAGCGTACAGGTGAACGTGTGACCGCCGAAGAGATTCGGTATGTAGCTTCTGAACTTGAAGATACTTTAGGTGG‑TGTCTACTCTATCCTTTCTCAAGAATTACAATTGTCTCTGGTACGAGTG‑CTCTTGAAGCAACTACAAGCCACGCAACAGATTCCTGAGTTACCTAAGGAAGCCGTAGAGCCAACCATTAGTACAGGTCTGGAAGCAATTGGTCGAGGAc                <  1:128536/300‑1 (MQ=255)`  
                                                                                                                                                                                                                                                                                               `ttACTGTAGCTAACGCCGTAAGTGACGCTATCGAGGCTCGCCTTTCGTTTGCCTTTATGTTGAACTCTGCGGTTCAGCGTACAGGTGAACGTGTGACCGCCGAAGAGATTCGGTATGTAGCTTCTGAACTTGAAGATACTTTAGGTGG‑TGTCTACTCTATCCTTTCTCAAGAATTACAATTGCCTCTGTTACGAGTT‑CTCTTGAAGCAACTACAAGCCACGCAACAGATTCCTGAGTTACCTAATGAAGCCGTAGAGCCAACCATTAGTCCAGTTCTGGAAGCAATTGGTCGGGTACAAGa            >  1:439244/1‑301 (MQ=255)`  
                                                                                                                                                                                                                                                                                                `tACTGCAGCTAACGTCGTAAGTGACGCTATCGAGGCTCGCCTTTCGTTTGCCTTGACGTTGAACTCTGCGGTTCAGCGTACAGGTGAACGTGTGACCGCCGAAGAGATTCGGTATGTCGCTTCTGAACTTGAAGATACTTTAGGTGG‑TGTCTACTATATCCTTTCTCAAGAATTACAATTGCCTCTGGTACGAGTG‑CTCTTGAAGCAACTACAAGCCACGCAACAGATTCCTGAGTTACCTAAGGAAGCCGTAGAGCCAACCATTAGTACAGGTCTGGAAGCAATTGGTCGAGGACAAGAc           <  1:21610/301‑1 (MQ=255)`  
                                                                                                                                                                                                                                                                                                    `gTAGCTAACTCCGTAAGTGACGCTATCGAGGCTCGCCTTTCGTTTGCCTTTATGTTGAACTCTGCGGTTCAGCGTACAGGTGAACGTGTGACCGCCGAAGAGATTCGGTATGTAGCTTCTGAACTTGAAGATACTTTAGGTGG‑TGTCTACTATATCCTTTCTCAAGAATTACAATTGCCTCTGGTACGAGTG‑CTCTTGAAGCAACTACAAGCCACGCAACAGATTCCTGAGTTACCTAAGGAAGCCGTAGAGCCAACCATTAGTACAGGTCTGGAAGCAATTGGTCGAGGACAAGACCttt       >  1:527225/1‑300 (MQ=255)`  
                                                                                                                                                                                                                                                                                                       `gCTAACGCCGTAAGAGACGGTATCGAGGCTCGCCTTTCATTTGCCGTTATGTTTAACTCTGCGGTTCAGCGTACAGGTTAACGTGTGAACGCCGAATAGATTCGGTATGTAGCTTCTGAACTTTAAGATACTTTAGGTGG‑TGTCTACTCTAGCCTTTCTAAAGAATTACAAGTGCCACTGTTACGAGTG‑CTCTTGAAGCAACTACAAGCCACGCAACAGATTCCTGAGTTACCTAAGGAAGCCGTAGAGCCAACCATTAGTACAGGTCTGGAAGCAATTGGTCGAGGACAAGACCTTGATa    <  1:209303/301‑1 (MQ=255)`  

                                                                                                                                                                                                                                                                                                            `|`                                                                                                                                                                                                                                                                                                             
`GACTTATCCTAAAGAGGCTTGCCCATACATCCCGATTCGGATGGTCAGACTAGATGGTGAATCCTACGGTCGTTCGTACATTGAGGAATACTTAGGTGACTTACGGTCCCTTGAAAATCTCCAAGAGGCTATCGTCAAGATGTCCATGATTAGCTCTAAGGTTATCGGCTTAGTGAATCCTGCTGGTATCACCCAGCCACGCCGACTGACCAAAGCTCAGACTGGTGACTTCGTTACTGGTCGTCCAGAAGACATCTCGTTCCTCCAACTGGAGAAGCAAGCAGACTTTACTGTAGCTAAAGCCGTAAGTGACGCTATCGAGGCTCGCCTTTCGTTTGCCTTTATGTTGAACTCTGCGGTTCAGCGTACAGGTGAACGTGTGACCGCCGAAGAGATTCGGTATGTAGCTTCTGAACTTGAAGATACTTTAGGTGG‑TGTCTACTCTATCCTTTCTCAAGAATTACAATTGCCTCTGGTACGAGTG‑CTCTTGAAGCAACTACAAGCCACGCAACAGATTCCTGAGTTACCTAAGGAAGCCGTAGAGCCAACCATTAGTACAGGTCTGGAAGCAATTGGTCGAGGACAAGACCTTGATAAG  >  NC_001604.1/20965‑21562`

  

Alignment Legend

Aligned base mismatch/match (shaded by quality score): `ATCG/ATCG` `< 3 ≤` `ATCG/ATCG` `< 10 ≤` `ATCG/ATCG` `< 21 ≤` `ATCG/ATCG` `< 34 ≤` `ATCG/ATCG` `< 38 ≤` `ATCG/ATCG`

Unaligned base: `atcg`    Masked matching base: `atcg`    Alignment gap: `‑`    Deleted base: `‑`
